# DFAST_QC: quality assessment and taxonomic identification tool for prokaryotic Genomes

**DOI:** 10.1186/s12859-024-06030-y

**Published:** 2025-01-07

**Authors:** Mohamed Elmanzalawi, Takatomo Fujisawa, Hiroshi Mori, Yasukazu Nakamura, Yasuhiro Tanizawa

**Affiliations:** 1 Department of Genetics, School of Life Science, The Graduate University for Advanced Studies (SOKENDAI), Mishima, 411-8540 Japan; 2 Department of Informatics, National Institute of Genetics, Mishima, 411-8540 Japan

**Keywords:** Taxonomy, Prokaryote, Database, INSDC, ANI

## Abstract

**Background:**

Accurate taxonomic classification in genome databases is essential for reliable biological research and effective data sharing. Mislabeling or inaccuracies in genome annotations can lead to incorrect scientific conclusions and hinder the reproducibility of research findings. Despite advances in genome analysis techniques, challenges persist in ensuring precise and reliable taxonomic assignments. Existing tools for genome verification often involve extensive computational resources or lengthy processing times, which can limit their accessibility and scalability for large-scale projects. There is a need for more efficient, user-friendly solutions that can handle diverse datasets and provide accurate results with minimal computational demands. This work aimed to address these challenges by introducing a novel tool that enhances taxonomic accuracy, offers a user-friendly interface, and supports large-scale analyses.

**Results:**

We introduce a novel tool for the quality control and taxonomic classification tool of prokaryotic genomes, called DFAST_QC, which is available as both a command-line tool and a web service. DFAST_QC can quickly identify species based on NCBI and GTDB taxonomies by combining genome-distance calculations using MASH with ANI calculations using Skani. We evaluated DFAST_QC's performance in species identification and found it to be highly consistent with existing taxonomic standards, successfully identifying species across diverse datasets. In several cases, DFAST_QC identified potential mislabeling of species names in public databases and highlighted discrepancies in current classifications, demonstrating its capability to uncover errors and enhance taxonomic accuracy. Additionally, the tool’s efficient design allows it to operate smoothly on local machines with minimal computational requirements, making it a practical choice for large-scale genome projects.

**Conclusions:**

DFAST_QC is a reliable and efficient tool for accurate taxonomic identification and genome quality control, well-suited for large-scale genomic studies. Its compatibility with limited-resource environments, combined with its user-friendly design, ensures seamless integration into existing workflows. DFAST_QC's ability to refine species assignments in public databases highlights its value as a complementary tool for maintaining and enhancing the accuracy of taxonomic data in genomic research. The web version is available at https://dfast.ddbj.nig.ac.jp/dqc/submit/, and the source code for local use can be found at https://github.com/nigyta/dfast_qc.

**Supplementary Information:**

The online version contains supplementary material available at 10.1186/s12859-024-06030-y.

## Background

Public genome databases are fundamental to biological research, where ensuring accurate metadata and high-quality sequences supports open data practices and facilitates collaborative research efforts. However, taxonomically mislabeled genomes within the databases can confuse or lead to scientifically inaccurate results when referenced or reused in other researches [1, 2].

To ensure accurate taxonomic labeling, the National Center for Biotechnology Information (NCBI) has used Average Nucleotide Identity (ANI) analysis since 2018 to verify prokaryotic genomes in GenBank [3]. ANI is a method that compares the genetic similarity between two genomes by calculating the mean identity of the homologous regions from the pairwise alignment between two genomes, with a threshold of 95% ANI commonly accepted to distinguish species [4]. Within the International Nucleotide Sequence Database Collaboration (INSDC), taxonomic information is organized based on NCBI Taxonomy to maintain the consistency and interoperability of the organism names [5]. The names of prokaryotes in NCBI Taxonomy are curated to best align with the authoritative nomenclature defined by the List of Prokaryotic Names with Standing in Nomenclature (LPSN) [6]. As such, NCBI Taxonomy serves as a standard, although not authoritative, resource for nomenclature and classification.

Apart from the efforts by database maintainers, accurate genome identification by users is equally crucial. As the number of genomes handled within a single research project increases, issues such as sample mix-ups, contamination, and misidentification of species that are difficult to distinguish using phylogenetic markers can arise. To avoid these potential pitfalls, it is recommended to conduct quality control of the genomes used in the project at the early stages of research. Existing tools, such as the Type Strain Genome Server (TYGS) [7] and the Microbial Genomes Atlas (MiGA) [8], are often equipped with large reference databases composed of numerous type strain genomes, leading to relatively long execution times and the necessity to offer them as web tools. This web-based nature, however, makes them less suitable for processing large numbers of genomes simultaneously. GTDB-Tk allows for the phylogenetic classification of genomes, even for uncultured microorganisms, on a local machine [9]. However, its high computational resource requirements make it challenging to run on smaller-scale computers. Additionally, the taxon names, based on its own taxonomic system called GTDB Taxonomy, may not necessarily align with the validly described names in LPSN or NCBI Taxonomy, which indicates that GTDB-Tk may not be well-suited for the validation of genomes to be deposited in sequence databases.

We have developed a genome verification tool DFAST_QC with an aim to ensure accurate taxonomic assignment and quality control of prokaryotic genomes. It is publicly available through the DFAST web service, which is a genome annotation and data submission pipeline for the Data Bank of Japan (DDBJ) [10]. It also functions as a standalone tool that can run on a local machine even with limited computational resources. DFAST_QC performs quick taxonomic identification based on NCBI Taxonomy using MASH [11] similarity estimation and Skani [12] for accurate ANI calculation. It also assesses genome completeness and contamination using CheckM [13]. It can optionally query against representative genomes in GTDB Taxonomy [14]. In this paper, we will present the features of DFAST_QC, focusing on taxonomic identification.

## Implementation

### Workflow of DFAST_QC

**Fig. 1:**
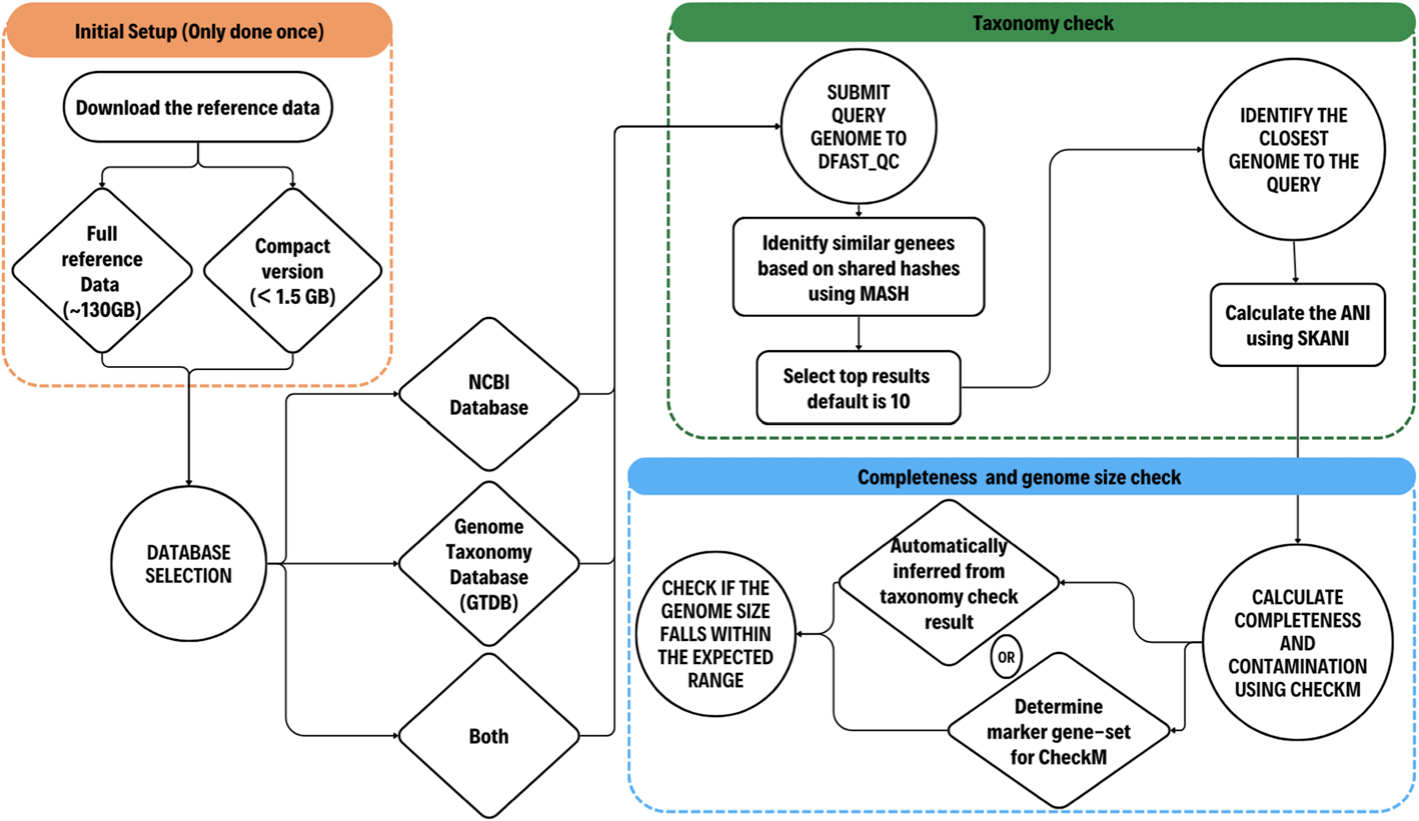
Workflow of DFAST_QC. It takes one or more genome sequence files in FASTA format as input, and performs taxonomic identification in a two-step approach using MASH and Skani, then conducts a genome completeness check using CheckM. Reference data for both NCBI Taxonomy and GTDB Taxonomy are available

DFAST_QC performs taxonomy checks using a two-step approach to reduce running time while maintaining accuracy. The required input is a simple FASTA file. Initially, the FASTA file undergoes genomic distance calculation using the MASH sketch file generated from reference genome sequences. In the second step, Skani is used to create a sketch file for these genomes, resulting in a manageable sketch file size and increased process speed. Then, ANI is calculated between the query genome and the selected reference genomes to determine taxonomic assignment, applying species-specific ANI thresholds when available, or using a default threshold of 95%. For the quality assessment, CheckM is employed to assess the completeness and contamination percentage of the query genome. The marker set for CheckM is automatically determined based on the result of the taxonomy check or can be specified manually. Finally, the genome size is checked to ensure it falls within the expected range. When specified as an option, species identification is performed based on GTDB Taxonomy by searching against its representative genomes. The overview of the workflow can be found in Fig. 1. An example use case can be found in Text S1 in the supplementary data.

### Preparation of the reference data

**Fig. 2:**
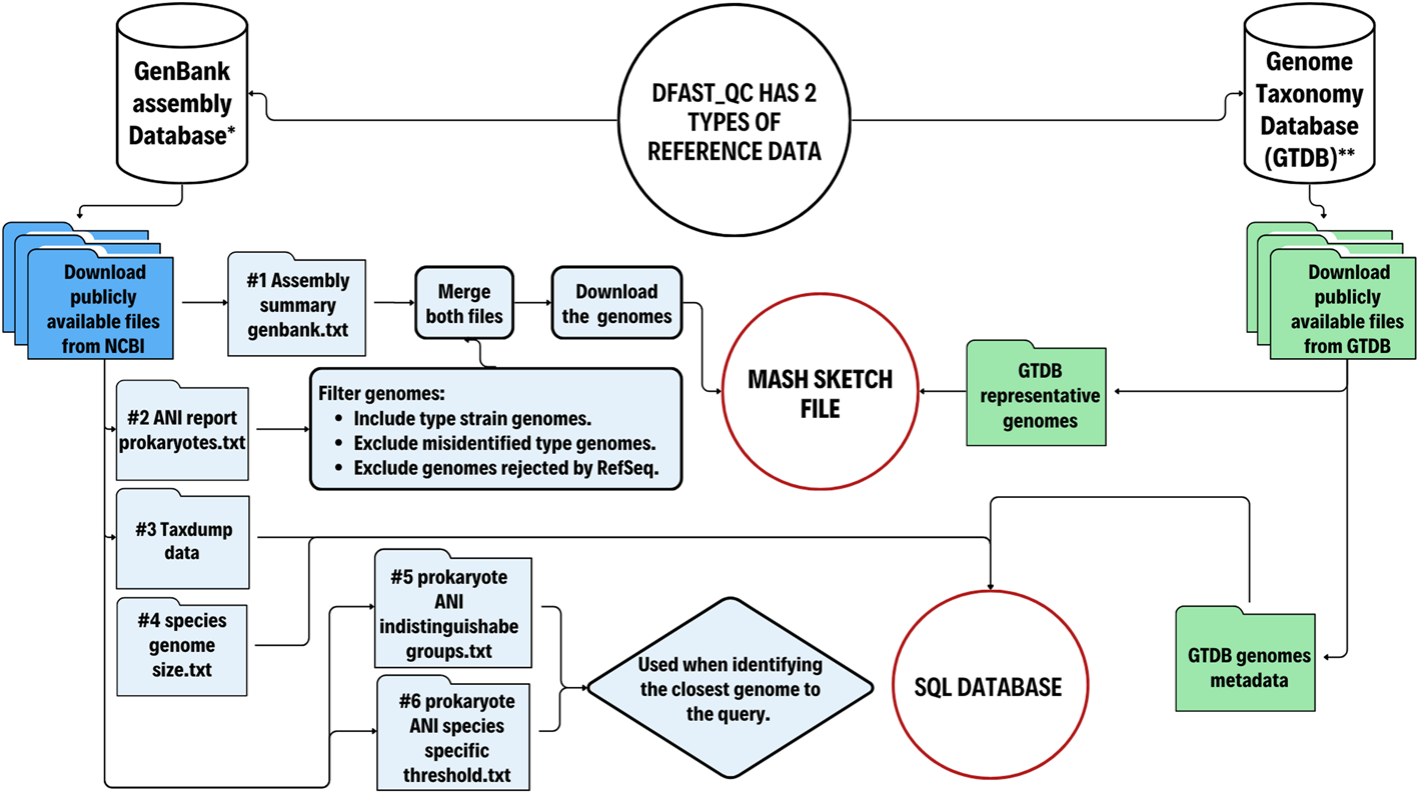
Schematic diagram of the procedures for preparing the reference data. All the files are retrieved from the NCBI FTP server (*https://ftp.ncbi.nlm.nih.gov/genomes/) and GTDB (**https://gtdb.ecogenomic.org/downloads)

DFAST_QC utilizes two primary sources of reference data: NCBI Datasets and GTDB. They can be accessed and managed using Python scripts included in the software package. Ready-to-use prebuilt reference data is also available to facilitate the initial setup. A detailed figure for data preparation can be found in Fig. 2.

#### Reference data for NCBI Taxonomy

DFAST_QC first retrieves metadata on genomic assemblies from GenBank (assembly_summary_genbank.txt) and identifies type strains (type genomes) from this dataset. Subsequently, it filters out genomes excluded from RefSeq or identified as misidentified type genomes, using criteria defined in the 'assembly type category', 'excluded from RefSeq', and 'taxonomy check status' columns within the NCBI-provided file (ANI_report_prokaryotes.txt). Following this, DFAST_QC proceeds to download the filtered genomes. Afterward, it creates an SQL database that integrates information from both”ANI_report_prokaryotes.txt” and”assembly_summary_genbank.txt”. Finally, DFAST_QC utilizes MASH to sketch the entire genomes and generate a consolidated sketch file. To identify the ANI threshold for each species and indistinguishable groups, DFAST_QC retrieves the “prokaryote ANI indistinguishable groups.txt” and “prokaryote ANI species specific_threshold.txt” files from NCBI.

#### Reference data for GTDB taxonomy

DFAST_QC downloads representative genomes and their metadata file from GTDB, and then it creates a dedicated SQL database optimized for searches within GTDB. Finally, it generates another sketch file using a similar methodology as described earlier.

### Benchmarking

To evaluate the performance of DFAST_QC, we conducted a series of comparative benchmarks. The reference data for DFAST_QC and benchmarking datasets were prepared on June 26, 2024. This reference data included 22,171 type genomes obtained from the NCBI Assembly Database and 113,104 representative genomes from GTDB release 220. Two benchmarking datasets, A and B, were prepared to evaluate the accuracy of species assignment based on the NCBI and GTDB taxonomies, respectively. Dataset A comprises 5184 of the latest non-type genomes, with one genome per species randomly selected from the NCBI GenBank. Based on NCBI’s quality control, we excluded species lacking available type genomes, those with failed or inconclusive taxonomy checks, and those deemed suppressed, or contaminated. Dataset B comprises 10,000 randomly selected metagenome-assembled genomes (MAGs) from the GEMs dataset [15].

Genomes in both Datasets A and B were processed by DFAST_QC (ver. 1.0.0) with default settings using a single CPU for each run. Genomes in Dataset B were also processed using the classify workflow (classify_wf) of GTDB-Tk version 2.4.0 with reference data release 220.

To evaluate the performance of DFAST_QC in terms of runtime and memory usage compared to other existing tools, 10 genomes were randomly selected from Dataset A and used as query inputs. DFAST_QC was run with taxonomic identification enabled, based on both NCBI and GTDB taxonomies. The classify workflow of GTDB-Tk was executed with default settings, and the same queries were submitted to the online services of TYGS and MiGA. Specifically, GTDB-Tk performs quick species identification using ANI (ANI screen) and higher-rank classification based on relative evolutionary distance (RED). Additionally, 10 genomes from Dataset B, for which the ANI screen failed, were randomly selected to evaluate the performance of classification based on RED.

### Web user interface

**Fig. 3:**
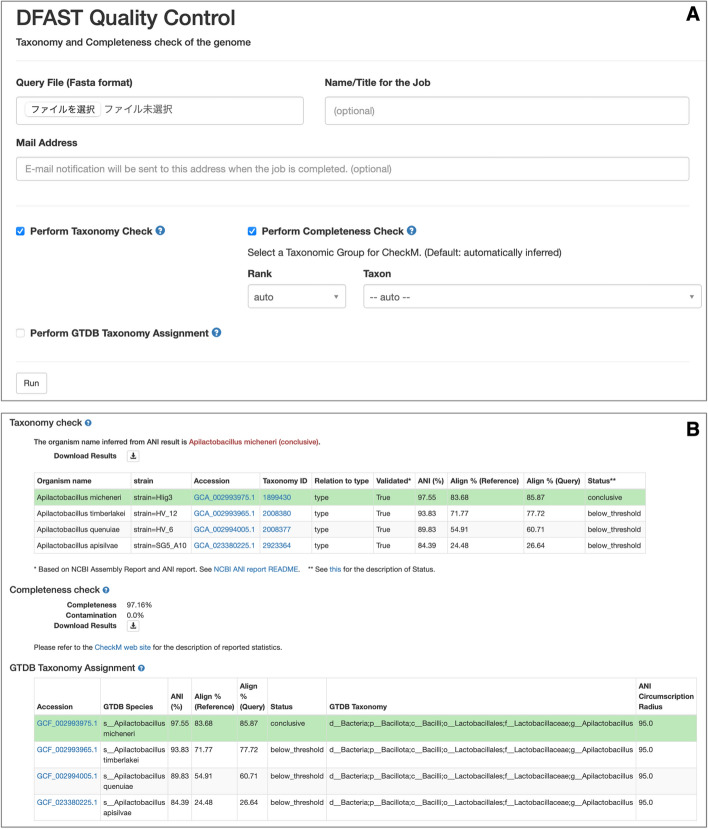
Screenshot of the web user interface of DFAST_QC. **A** Job submission form. Users can upload a query genome file in a FASTA format to perform taxonomy check based on NCBI Taxonomy, species identification based on GTDB Taxonomy, and completeness check using CheckM. **B** Example of the result page. ANI values against closely-related reference genomes are shown with the accepted hits (ANI ≥ threshold) highlighted. The completeness and contamination values are also shown on the same page

The web version of DFAST_QC is available as part of the DFAST web service of DDBJ. The system was implemented using Python and the Flask web framework. It is accessible without any user registration, and the reference data is updated regularly. Figure 3 shows the screenshots of the job submission form and the result page. The DFAST_QC pipeline can also be enabled as an option for the genome annotation pipeline of the web service.

## Results and discussion

### Benchmarking based on NCBI taxonomy

The accuracy of species assignments based on NCBI Taxonomy was assessed using Dataset A, which comprises a collection of randomly selected non-type genomes from GenBank. We compared the species names assigned by DFAST_QC using the top ANI hit to the species name as labeled for the submitted genome in GenBank (declared species). The results of this comparison are summarized in Table 1.

**Table 1:** DFAST_QC results for 5,184 non-type genomes from GenBank

DFAST_QC species classification*	Comparison with declared species name in GenBank		Total
	Match	Mismatch	
Conclusive	4664	2	4666
Inconclusive	504	0	504
Indistinguishable	12	1	13
No hit	0	1	1
Total	5180	4	5184

*Conclusive: accepted hits (ANI > threshold) only against a single species, Inconclusive: hits (ANI > threshold) against multiple species, Indistinguishable: hits against indistinguishable groups, e.g., *Escherichia coli* and *Shigella* spp

Out of the 5184 cases compared, the species name assigned by DFAST_QC matched the declared species in GenBank in 5180 cases (99.9%), including 504 cases with accepted ANI hits (ANI > threshold) against multiple species and 12 cases that fell into indistinguishable species groups. The four mismatch cases were likely caused by the mislabeling of the declared species, the misclassification of reference genomes, or cases of misidentification within closely related species, as summarized in Table 2. These examples raise ongoing issues, such as the presence of mislabeled genomes despite efforts in species name validation within databases, and the need for reclassification in certain groups. Further details can be found in Text S2 in the supplementary data. For many of the inconclusive cases, the declared species names were found in the top hit (416 cases) or in the second or lower hits with an ANI value slightly below the threshold (88 cases), indicating that they belonged to species difficult to differentiate by ANI or might be an outlier within the species.

**Table 2:** The 4 mismatch cases resulted from the NCBI taxonomy benchmark

Declared species	NCBI accession	DFAST_QC result	ANI value	Mismatch explanation
*Actinosynnema pretiosum*	GCA_002354875.1	*Actinosynnema mirum*	96.65	Possible misclassification of the type strain (*A. pretiosum* subsp*. auranticum* DSM 44131^T^), indicating the need for reclassification
*Zymomonas mobilis* subsp*. mobilis*	GCA_000576125.1	*None*	–	Possible misclassification of the type strain (*Z. mobilis* subsp. *mobilis* ATCC 10988^T^), indicating the need for reclassification
*Lactobacillus gasseri*	GCA_027152945.1	*Lactobacillus paragasseri*	98.35	Mislabeling of the query genome. The result of DFAST_QC supports the recent reclassification of the species
*Shigella dysenteriae*	GCA_013997415.1	*Shigella boydii*	99.68	Misidentification within indistinguishable species

### Benchmarking based on GTDB Taxonomy

Species-level taxonomy classification based on GTDB Taxonomy was evaluated by comparing the results from DFAST_QC and GTDB-Tk (Table 3). The benchmarking using Dataset B showed high consistency with the results from GTDB-Tk at the species-level identification.

**Table 3:** Comparison of species-level classification for 10,000 MAGs from GEMs between DFAST_QC and GTDB-Tk

DFAST_QC species classification	GTDB-Tk species classification		Total
	Assigned	Unassigned	
Assigned	Identical/7056	33	7157
	Mismatch/68		
Unassigned	2	2841	2843
Total	7126	2874	10,000

"Assigned": Genomic sequences classified into specific taxonomic species with ANI > 95%. "Unassigned": Genomic sequences that could not be classified

DFAST_QC successfully assigned species names to 7124 genomes, with 7056 (99%) of these classifications matching those made by GTDB-Tk. The remaining 68 genomes exhibited mismatches in species classification, primarily due to multiple candidate genomes with ANI values around 95% or closely similar ANI values, highlighting the challenges in precise species identification.

## General discussion

The benchmark demonstrates DFAST_QC's accuracy in species identification when a reference type genome is available. However, for species lacking a sequenced type genome, DFAST_QC cannot definitively assign species. According to the NCBI report (prokaryote_without_type_assembly.txt), at least 2500 species currently lack sequenced type genomes, even though at least one non-type genome is deposited in GenBank [16]. Fortunately, this situation is improving thanks to large sequencing projects like the Global Catalog of Microorganisms (GCM) 10 K type strain genome sequencing project [17, 18], and the growing recommendation to deposit genome sequences alongside new taxon descriptions [19]. Also, as exemplified in the four mismatch cases of Dataset A, the functionality to search against GTDB representative genomes can serve as a complement to the results of taxonomy checks, particularly when reference genomes are not available.

Table 4 shows the comparison between DFAST_QC, GTDB-Tk, MiGA, and TYGS in terms of runtime, memory usage, and functionality. The evaluation was conducted using 10 randomly selected genomes from Datasets A and B. For canonical taxonomic identification, comparison should be made against reference data derived from type strains. Since TYGS and MiGA are only accessible online, DFAST_QC is, to the best of our knowledge, the only tool capable of accomplishing this on local machines. Unlike other genome-based identification tools DFAST_QC's results are limited to species-level identification, with no phylogenetic inference at higher taxonomic ranks. This is because our focus is more on the correct assignment of organism names for genomes to be submitted to public sequence databases. Due to its simplicity, DFAST_QC operates on machines with limited computational resources. In fact, it requires less than 2 GB of memory and can typically complete taxonomy identification within 10 s, which is significantly faster and more memory-efficient than the quick species identification performed by GTDB-Tk's ANI screen. Additionally, we provide a minimal set of prebuilt reference data containing only sketch files and metadata (< 1.5 GB in size). Although this approach results in extra execution time since reference genomes required for ANI calculation are retrieved on-the-fly during runtime, it streamlines the installation process on local machines by eliminating the need to prepare a full set of reference data (~ 130 GB, including approx. 100 GB of GTDB representative genomes). This balance of performance and simplicity makes DFAST_QC a practical and user-friendly choice. Being simple and lightweight also makes integration into other analytical workflows easier.

**Table 4:** Comparison between DFAST_QC, TYGS, MiGA, and GTDB-Tk

	DFAST_QC	TYGS	MiGA	GTDB-Tk
Run time	7.8 s (identification only) 2m 58s (incl. completeness check)	30–180 m*	5–10 m*	30.5 s (ANI) 20m0s (RED)
Memory usage	0.8G (identification only) 1.8G (incl. completeness check)	n.a	n.a	33.4G (ANI) 161.9G (RED)
Availability	Web/CL	Web	Web	CL**
Reference Data	Type strains / GTDB	Type strains	Type strains	GTDB
Species identification method	ANI	dDDH	ANI	ANI
Classification above species level	✕	✕	AAI	RED
Completeness/Contamination check	✓	✕	✓	✕
Genome size check	✓	✕	✕	✕
Other features	Both full/compact-sized reference data are available	Phylogenetic tree	Gene prediction	de_novo_wf***
Citation	This study	[7]	[8]	[9]

*The run time may depend on the server workload

**The Online version is also at KBase https://www.kbase.us

***Construction of new phylogenomic trees including user-provided genomes

Finally, we would like to reiterate the critical importance of public sequence databases in microbial taxonomy. In modern microbial classification and identification, sequence-based methods have become standard practice for both eukaryotic and prokaryotic organisms. Specifically, nucleotide sequences derived from type material serve as essential references, with significant efforts devoted to their curation and validation to ensure consistency and reliability [16, 20]. These data are publicly accessible and freely reusable, making them invaluable resources for a wide range of analyses, including taxonomy and comparative genomics. DFAST_QC enhances access to these reference data through a user-friendly web service and a simple command-line interface, enabling researchers to validate their own data effectively. Given that nucleotide sequence databases function as archives for research data, the quality and accuracy of the submitted data and metadata must be the responsibility of the submitters. Although database maintainers perform validation checks on submitted data, the sheer volume of submissions necessitates reliance on automated processes for a significant portion of this validation. Therefore, the role of tools like DFAST_QC becomes increasingly vital, allowing researchers to ensure the integrity of their data before submission, thereby contributing to the overall quality of public sequence databases.

## Conclusions

DFAST_QC is a tool designed for quality and taxonomy check of prokaryotic genomes, utilizing NCBI and GTDB taxonomies for species identification. It is integrated into the web service of DDBJ's genome annotation and submission pipeline, DFAST, featuring a user-friendly interface for researchers unfamiliar with the command-line operation. In addition, it is also available as a stand-alone software, which enables rigorous validation of genomes on a local machine before submission to public databases. It employs compact reference data and requires low computational resources. This comprehensive functionality reinforces its importance in maintaining the accuracy and reliability of genomic data across scientific research.

### Availability and requirements

**Project name:** DFAST_QC

**Project home page:** https://github.com/nigyta/dfast_qc

**Operating system(s):** Linux and Linux-like operating systems including MacOS and Windows WSL2

**Programming language:** Python

**Other requirements:** Python 3.9 or higher

**License:** GPLv3

**Any restrictions to use by non-academics:** None

## Supplementary Information

Additional file 1

Additional file 2

## Data Availability

DFAST_QC is available both as a web service (https://dfast.ddbj.nig.ac.jp/dqc) and as a stand-alone command line tool. The source code is available under the GPLv3 license at: https://github.com/nigyta/dfast_qc, and the conda package is also available from Bioconda. The data and scripts used for the benchmarking process are publicly available on GitHub (https://github.com/Mohamed-Elmanzalawi/DFAST_QC_Benchmark).

## Abbreviations

NCBI: National Center for Biotechnology Information
GTDB: Genome Taxonomy Database
ANI: Average Nucleotide Identity
AAI: Average Amino Acid Identity
dDDH: Digital DNA–DNA Hybridization
RED: Relative Evolutionary Distance
INSDC: International Nucleotide Sequence Database Collaboration
LPSN: List of Prokaryotic Names with Standing in Nomenclature
TYGS: Type Strain Genome Server
MiGA: Microbial Genomes Atlas
GTDB-Tk: Genome Taxonomy Database Toolkit
DDBJ: DNA Data Bank of Japan
DFAST: DDBJ Fast Annotation and Submission Tool
MAGs: Metagenome-Assembled Genomes
GEMs: Genomes from Earth’s Microbiomes

## References

[1] Bagheri H, Severin AJ, Rajan H. Detecting and correcting misclassified sequences in the large-scale public databases. Bioinformatics. 2020;36:4699–705. 32579213 10.1093/bioinformatics/btaa586 PMC7821992

[2] Goudey B, Geard N, Verspoor K, Zobel J. Propagation, detection and correction of errors using the sequence database network. Brief Bioinform. 2022;23:bbac416. 36266246 10.1093/bib/bbac416 PMC9677457

[3] Ciufo S, Kannan S, Sharma S, Badretdin A, Clark K, Turner S, et al. Using average nucleotide identity to improve taxonomic assignments in prokaryotic genomes at the NCBI. Int J Syst Evol Microbiol. 2018;68:2386–92. 29792589 10.1099/ijsem.0.002809 PMC6978984

[4] Goris J, Konstantinidis KT, Klappenbach JA, Coenye T, Vandamme P, Tiedje JM. DNA-DNA hybridization values and their relationship to whole-genome sequence similarities. Int J Syst Evol Microbiol. 2007;57(Pt 1):81–91. 17220447 10.1099/ijs.0.64483-0

[5] Schoch CL, Ciufo S, Domrachev M, Hotton CL, Kannan S, Khovanskaya R, et al. NCBI Taxonomy: a comprehensive update on curation, resources and tools. Database J Biol Databases Curation. 2020;2020:baaa062. 10.1093/database/baaa062 PMC7408187 32761142

[6] Meier-Kolthoff JP, Carbasse JS, Peinado-Olarte RL, Göker M. TYGS and LPSN: a database tandem for fast and reliable genome-based classification and nomenclature of prokaryotes. Nucleic Acids Res. 2022;50:D801–7. 34634793 10.1093/nar/gkab902 PMC8728197

[7] Meier-Kolthoff JP, Göker M. TYGS is an automated high-throughput platform for state-of-the-art genome-based taxonomy. Nat Commun. 2019;10:2182. 31097708 10.1038/s41467-019-10210-3 PMC6522516

[8] Rodriguez-R LM, Gunturu S, Harvey WT, Rosselló-Mora R, Tiedje JM, Cole JR, et al. The microbial genomes atlas (MiGA) webserver: taxonomic and gene diversity analysis of Archaea and Bacteria at the whole genome level. Nucleic Acids Res. 2018;46:W282–8. 29905870 10.1093/nar/gky467 PMC6031002

[9] Chaumeil P-A, Mussig AJ, Hugenholtz P, Parks DH. GTDB-Tk v2: memory friendly classification with the genome taxonomy database. Bioinformatics. 2022;38:5315–6. 36218463 10.1093/bioinformatics/btac672 PMC9710552

[10] Tanizawa Y, Fujisawa T, Nakamura Y. DFAST: a flexible prokaryotic genome annotation pipeline for faster genome publication. Bioinformatics. 2018;34:1037–9. 29106469 10.1093/bioinformatics/btx713 PMC5860143

[11] Ondov BD, Treangen TJ, Melsted P, Mallonee AB, Bergman NH, Koren S, et al. Mash: fast genome and metagenome distance estimation using MinHash. Genome Biol. 2016;17:132. 27323842 10.1186/s13059-016-0997-x PMC4915045

[12] Shaw J, Yu YW. Fast and robust metagenomic sequence comparison through sparse chaining with skani. Nat Methods. 2023;20:1661–5. 37735570 10.1038/s41592-023-02018-3 PMC10630134

[13] Parks DH, Imelfort M, Skennerton CT, Hugenholtz P, Tyson GW. CheckM: assessing the quality of microbial genomes recovered from isolates, single cells, and metagenomes. Genome Res. 2015;25:1043–55. 25977477 10.1101/gr.186072.114 PMC4484387

[14] Parks DH, Chuvochina M, Rinke C, Mussig AJ, Chaumeil P-A, Hugenholtz P. GTDB: an ongoing census of bacterial and archaeal diversity through a phylogenetically consistent, rank normalized and complete genome-based taxonomy. Nucleic Acids Res. 2022;50:D785–94. 34520557 10.1093/nar/gkab776 PMC8728215

[15] Nayfach S, Roux S, Seshadri R, Udwary D, Varghese N, Schulz F, et al. A genomic catalog of Earth’s microbiomes. Nat Biotechnol. 2021;39:499–509. 33169036 10.1038/s41587-020-0718-6 PMC8041624

[16] Kannan S, Sharma S, Ciufo S, Clark K, Turner S, Kitts PA, et al. Collection and curation of prokaryotic genome assemblies from type strains at NCBI. Int J Syst Evol Microbiol. 2023;73: 005707. 36748495 10.1099/ijsem.0.005707 PMC10228379

[17] Wu L, Ma J. The Global Catalogue of Microorganisms (GCM) 10K type strain sequencing project: providing services to taxonomists for standard genome sequencing and annotation. Int J Syst Evol Microbiol. 2019;69:895–8. 30832757 10.1099/ijsem.0.003276

[18] Shi W, Sun Q, Fan G, Hideaki S, Moriya O, Itoh T, et al. gcType: a high-quality type strain genome database for microbial phylogenetic and functional research. Nucleic Acids Res. 2021;49:D694-705. 33119759 10.1093/nar/gkaa957 PMC7778895

[19] Riesco R, Trujillo ME. Update on the proposed minimal standards for the use of genome data for the taxonomy of prokaryotes. Int J Syst Evol Microbiol. 2024;74:006300. 38512750 10.1099/ijsem.0.006300 PMC10963913

[20] Renner SS, Scherz MD, Schoch CL, Gottschling M, Vences M. Improving the gold standard in NCBI GenBank and related databases: DNA sequences from type specimens and type strains. Syst Biol. 2024;73:486–94. 37956405 10.1093/sysbio/syad068 PMC11502950

