# Supplementary material for: DFAST_QC: quality assessment and taxonomic identification tool for prokaryotic Genomes
Source: BMC Bioinformatics. 2025 Jan 7;26:3. doi: 10.1186/s12859-024-06030-y (PMC11705978; doi:10.1186/s12859-024-06030-y)
Supplement: Supplementary file 1 — Additional file 1 [file 12859_2024_6030_MOESM1_ESM.pdf]

Supplementary Information
DFAST_QC: quality assessment and taxonomic identification tool for prokaryotic Genomes

Mohamed Elmanzalawi[1], Takatomo Fujisawa[2], Hiroshi Mori[1, 2], Yasukazu Nakamura[1, 2], Yasuhiro Tanizawa[1, 2]

[1]Department of Genetics, School of Life Science, The Graduate University for Advanced Studies (SOKENDAI), Mishima 411-8540, Japan

[2]Department of Informatics, National Institute of Genetics, Mishima 411-8540, Japan

---

Contents:

Text S1. Example Use Case.. 2

Text S1.1 Using the command line... 2

Text S1.2 Using the web version... 3

Figure S1.DFAST_QC web interface:..3

Text S2. Details for the four mismatch cases in Dataset A...4

1. Actinosynnema pretiosum (GCA_002354875.1)... 4

2. Zymomonas mobilis subsp. mobilis (GCA_000576125.1)......................................5

3. Lactobacillus gasseri (GCA_027152945.1).. 6

4. Shigella dysenteriae (GCA_013997415.1).. 6

References.. 7

Text S1. Example Use Case

DFAST_QC is an open-source package written in Python, available as both source code and a web version. It was designed to be user-friendly, allowing users to generate accurate and easily interpreted results with minimal effort. To demonstrate the practicality and versatility of DFAST_QC, we present a use case involving the publicly available genome of *Paucilactobacillus hokkaidonensis* [1] on NCBI (GCA_000829395.1), which is included in the "`examples`" directory under the path where the DFAST_QC is installed.

Text S1.1 Using the command line

DFAST_QC can be downloaded from our GitHub repository at https://github.com/nigyta/dfast_qc. Additionally, it is also available on Bioconda [2]. After downloading the reference data, users can execute the following command: "`dfast_qc -i examples/GCA_000829395.1.fna.gz.fa -o result --enable_gtdb`".

DFAST_QC must be provided with a FASTA file to run. This file path is passed to the script through the "`–i`" argument. The results are stored in a directory named after the "`-o`" argument. If omitted, the results will be saved in a directory named 'OUT'. By default, DFAST_QC performs taxonomy classification on the NCBI Taxonomy. The optional parameter "`--enable_gtdb`" allows users to perform species identification using the representative genomes of GTDB. To search exclusively in the GTDB database, the optional command "`—-disable_tc`" can be used to disable the NCBI Taxonomy search. Another optional argument is "`-a`", which is an

integer and sets the minimal ANI threshold for genomes to be included in the analysis. This filter is useful when the user aims to identify highly related genomes. The default value is set to 95%.

Text S1.2 Using the web version

To provide convenient and rapid access to DFAST_QC for researchers preferring a graphical user interface, we have developed a web interface at https://dfast.ddbj.nig.ac.jp/dqc/. Users can upload their files directly and receive results promptly. Additionally, the reference gene marker set for CheckM can be specified; if unspecified, it is automatically selected based on the result of species identification. Users can enter their e-mail address and a job name, to receive an e-mail after the job is completed and identify different submissions easily. The result will be deleted 30 days after the last visit.

Figure S1.DFAST_QC web interface:

The figure shows the main sections for file upload, parameter settings, and user input fields.

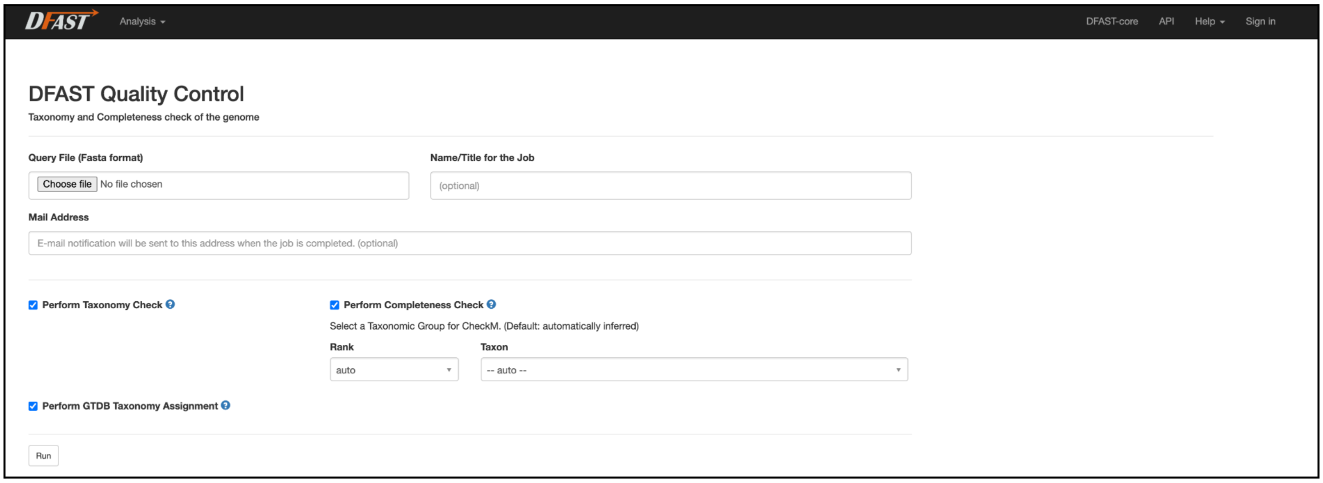

Text S2. Details for the four mismatch cases in Dataset A

The taxonomic mismatches identified during the benchmarking analysis underscore significant challenges in genome-based identification. These cases reveal discrepancies arising from misclassified type strains, ambiguous species definitions, and database inconsistencies. Below, we discuss each case in detail and explore their taxonomic implications.

1. *Actinosynnema pretiosum* (GCA_002354875.1)

The genome GCA_002354875.1, labeled as *Actinosynnema pretiosum* by the data submitter, was identified as *Actinosynnema mirum* by DFAST_QC with an ANI of 96.65%. Whereas by the result of NCBI's taxonomy check, it was identified as *A. pretiosum* based on the ANI value of 99.99% against its type strain (GCA_013387285.1, *A. pretiosum* subsp. *auranticum* DSM 44131^{T}). However, GCA_013387285.1 failed to match another type strain (GCA_024171695.1, A. *pretiosum* subsp. *pretiosum* DSM 44132^{T}) on the same species with 93.89% ANI, while showing the ANI value of 95.95% against GCA_000023245.1 (*Actinosynnema mirum*). This implies that *A. pretiosum* subsp. *auranticum* Hasegawa et al. 1983 may represent a later heterotypic synonym of *A. mirum* Hasegawa et al. 1978. Since the "Inconclusive" status is assigned to GCA_013387285.1 by NCBI's check, we excluded it from our reference data. This is why our result, in which GCA_002354875.1 was identified as *A. mirum*, was incongruent with that of NCBI's check. Notably, both GCA_002354875.1 and GCA_013387285.1 are elevated into a species-level cluster named *'Actinosynnema auranticum'* in GTDB Taxonomy r220, forming a clade distinct from *A. pretiosum* and *A. mirum*.

(https://www.ncbi.nlm.nih.gov/datasets/genome/GCA_002354875.1/, https://www.ncbi.nlm.nih.gov/datasets/genome/GCA_013387285.1/, https://gtdb.ecogenomic.org/species?id=Actinosynnema%20auranticum accessed on 2024/7/1).

2. *Zymomonas mobilis* subsp. *mobilis* (GCA_000576125.1)

The genome GCA_000576125.1 failed to produce an accepted hit in DFAST_QC. It was due to a reason similar to the case of GCA_002354875.1. The type genome of this subspecies (GCA_000175255.2, *Zymomonas mobilis* subsp. *mobilis* ATCC 10988^{T}) was assigned an "Inconclusive" status by NCBI's check due to its low ANI (80.41%) against the type genome of the other subspecies (GCA_006539385.1, *Zymomonas mobilis* subsp. *pomaceae* NBRC 13757^{T}). Therefore, GCA_006539385.1 was excluded from the reference data of DFAST_QC. The two subspecies should ideally be reclassified into two distinct species. In fact, in GTDB Taxonomy, they are placed in two different species-level clades, *Z. mobilis* and *Z. pomaceae*. Accordingly, GCA_000576125.1 was classified as '*Zymomonas mobilis*' when DFAST_QC was executed using GTDB representative genomes as reference.

(https://www.ncbi.nlm.nih.gov/datasets/genome/GCA_000576125.1/ , https://www.ncbi.nlm.nih.gov/datasets/genome/GCA_000175255.2/, accessed on 2024/7/1).

3. *Lactobacillus gasseri* (GCA_027152945.1)

The genome GCA_027152945.1, labeled as *Lactobacillus gasseri*, was identified as *Lactobacillus paragasseri* with an ANI of 98.35% by DFAST_QC. This aligns with recent studies proposing *L. paragasseri* as a distinct species, having been separated from *L. gasseri* [3]. Many genomes currently labeled as *L. gasseri* in public databases likely belong to *L. paragasseri*, as reported in the prior research [4]. Considering these situations, GCA_027152945.1 should correctly be labeled as *L. paragasseri* to reflect recent taxonomic changes.

4. *Shigella dysenteriae* (GCA_013997415.1)

The genome GCA_013997415.1, labeled as *Shigella dysenteriae*, was found in a group that is difficult to distinguish by ANI (indistinguishable group) according to NCBI's "prokaryote_ANI_indistinguishable_groups.txt". While DFAST_QC identified it as *Shigella boydii* with an ANI of 97.5%, this value was below the species-specific threshold of 99.2%, resulting in classification ambiguity. However, it is worth mentioning that, since we limited our Mash top hit results to 10, *Shigella dysenteriae* was not included in our analysis. When we increased the threshold, it appeared with ANI 97.5%, although it was lower than its ANI species-specific threshold (99.2%). This case exemplifies the challenges of whole-genome based identification for organisms in specific groups, such as *Shigella* and *E. coli*, even when species-specific thresholds are provided.

References

1. Tanizawa Y, Tohno M, Kaminuma E, Nakamura Y, Arita M. Complete genome sequence and analysis of Lactobacillus hokkaidonensis LOOC260(T), a psychrotrophic lactic acid bacterium isolated from silage. BMC Genomics. 2015;16:240.

2. Grüning B, Dale R, Sjödin A, Chapman BA, Rowe J, Tomkins-Tinch CH, et al. Bioconda: sustainable and comprehensive software distribution for the life sciences. Nat Methods. 2018;15:475–6.

3. Tanizawa Y, Tada I, Kobayashi H, Endo A, Maeno S, Toyoda A, et al. Lactobacillus paragasseri sp. nov., a sister taxon of Lactobacillus gasseri, based on whole-genome sequence analyses. Int J Syst Evol Microbiol. 2018;68:3512–7.

4. Ene A, Stegman N, Wolfe A, Putonti C. Genomic insights into Lactobacillus gasseri and Lactobacillus paragasseri. PeerJ. 2022;10:e13479.
